# Detecting possibly frequent change-points: Wild Binary Segmentation 2 and steepest-drop model selection—rejoinder

**DOI:** 10.1007/s42952-020-00085-2

**Published:** 2020-09-16

**Authors:** Piotr Fryzlewicz

**Affiliations:** Department of Statistics, London School of Economics, Houghton Street, London, WC2A 2AE UK

**Keywords:** Segmentation, Break detection, Jump detection, randomized algorithms, Adaptive algorithms, Multiscale methods

## Abstract

Many existing procedures for detecting multiple change-points in data sequences fail in frequent-change-point scenarios. This article proposes a new change-point detection methodology designed to work well in both infrequent and frequent change-point settings. It is made up of two ingredients: one is “Wild Binary Segmentation 2” (WBS2), a recursive algorithm for producing what we call a ‘complete’ solution path to the change-point detection problem, i.e. a sequence of estimated nested models containing $0, \ldots , T-1$ change-points, where *T* is the data length. The other ingredient is a new model selection procedure, referred to as “Steepest Drop to Low Levels” (SDLL). The SDLL criterion acts on the WBS2 solution path, and, unlike many existing model selection procedures for change-point problems, it is not penalty-based, and only uses thresholding as a certain discrete secondary check. The resulting WBS2.SDLL procedure, combining both ingredients, is shown to be consistent, and to significantly outperform the competition in the frequent change-point scenarios tested. WBS2.SDLL is fast, easy to code and does not require the choice of a window or span parameter.

I would like to start by thanking all sets of discussants: Moulinath Banerjee; Haeran Cho and Claudia Kirch; Solt Kovács, Housen Li and Peter Bühlmann; Robert Lund and Xueheng Shi; and Myung Hwan Seo, for their extremely high-quality contributions. Without exception, I found each discussion contribution immensely thought-provoking, and each prompted an “I haven’t thought of this” reaction.

Some of the contributions contain overlapping themes, so I arrange my response thematically, rather than separating it by authors.

*Does WBS2.SDLL work for theoretically small spacings between change-points?* Both Banerjee and Cho and Kirch raise the issue of whether WBS2.SDLL works for settings in which spacings between change-points are *o*(*T*), in addition to the *O*(*T*) case discussed in the paper. After all, WBS2.SDLL is advertised as a procedure that works well in frequent change-point scenarios, so should not we expect this to be reflected in the fact that it permits *o*(*T*) spacings?

The answer here is that yes, WBS2.SDLL permits a range of *o*(*T*) spacings, but, in the version as presented in the paper, it will not theoretically permit the shortest possible spacings, for the following reason. As we know e.g., from the correction to Lemma 2 from Cho and Fryzlewicz (2012), available on https://people.maths.bris.ac.uk/~mahrc/papers/ubs_correction.pdf, and also from Kovács et al. (2020), Wild Binary Segmentation [(WBS, Fryzlewicz (2014)] does not estimate change-point locations, in *o*(*T*) spacing settings, as accurately as e.g., the Narrowest-Over-Threshold (NOT) procedure of Baranowski et al. (2019). Fundamentally, this is because WBS always selects the largest available CUSUMs, which may not be the best localisers. WBS2 inherits this suboptimal localisation property from WBS. A consequence of this fact is that WBS and WBS2 are more restrictive, in terms of permitted spacings between change-points, than NOT (the latter is near-optimal in this regard).

However, there may well be an interesting fix to this suboptimal localisation property of WBS and WBS2. Consider an alternative solution path procedure, termed ‘Sharp WBS’ (or: ‘Sharp WBS2’, depending on the interval sampling used), which only accepts CUSUMs with sufficiently sharp drops to the left and to the right off their maxima. Informally, let us refer to such CUSUMs as ‘sharp’. We know that sharp CUSUMs exist as long as there are still undetected change-points in the data, as CUSUMs computed over portions of the data that contain single change-points must be viewed as sharp. As sharp CUSUMs are the best possible localisers, the Sharp WBS(2) solution path procedure should be expected to be optimal, or near-optimal, in terms of change-point localisation, just like NOT. Note that while WBS(2) and NOT can be placed at two extremes in the sense that WBS(2) goes for the largest available CUSUMs and NOT for the smallest (significant) CUSUMs, Sharp WBS(2) could be placed ‘between’ WBS(2) and NOT, as it targets smaller CUSUMs than WBS(2) but larger than NOT.

If the sharp CUSUMs can be defined in such a way that they satisfy Lemma 2 from Cho and Fryzlewicz (2012), which should be perfectly possible, then working with sharp CUSUMs would amount to treating Lemma 2 as correct, rather than having to rely on correcting it, as was done in https://people.maths.bris.ac.uk/~mahrc/papers/ubs_correction.pdf. This point of view hopefully makes it easy to see why restricting ourselves to sharp CUSUMs would likely resolve the suboptimal localisation property of WBS(2).

However, working with Sharp WBS(2) as a solution path device might weaken the appeal of SDLL as a model selection criterion. The reason is that working with maximum available CUSUMs, as done in the original WBS(2) (even if they are not the best localisers) is beneficial for SDLL as it is likely to maximise the drop between the significant CUSUMs and the insignificant ones, which facilitates the correct operation of SDLL. If we were to work with the Sharp WBS(2), the resulting sharp CUSUMs would not necessarily be the largest available, which would likely reduce the drop for SDLL to detect, thereby possibly reducing the accuracy of SDLL as a model selector.

Therefore, from the point of view of WBS2.SDLL, there appears to be a trade-off between estimating the number of change-points and their locations. A WBS(2) solution path is better that a Sharp WBS(2) one for estimating the number of change-points via SDLL, and the opposite is true for estimating their locations. A possible compromise could be to use WBS2.SDLL for estimating the number of change points, and then Sharp WBS2.SDLL for estimating their locations.

*Random character of WBS2 and whether this poses a problem.* Both Cho and Kirch and Kovács et al. are critical of the fact that WBS2 uses a random interval sampling scheme; Cho and Kirch refer to MOSUM as “more systematic” than WBS2, and Kovács et al. mention that “seeded intervals lead to reproducible results unlike WBS2.SDLL”. I should point out that WBS(2) can by all means be based on a deterministic grid, and this would pose no problems whatsoever from the algorithmic or theoretical point of view. In WBS2, a deterministic analogue of random sub-interval sampling with uniformly distributed start- and end-points on a generic interval [*e*, *s*] would be to select all intervals $[s_m, e_m]$ such that $s_m, e_m \in \{ s, [s + \delta (e-s)], [s + 2\delta (e-s)], \ldots , e \}$, where $\delta \in [0,1]$ is chosen in such a way that the number of the thus-constructed intervals $[s_m, e_m]$ is the smallest integer larger than or equal a certain prescribed $\tilde{M}$. Equipping WBS(2) with a deterministic grid such as this one would make it “systematic” in the understanding of Cho and Kirch and lead to “reproducible results” in the sense of Kovács et al.

In view of the “lack of reproducibility” associated with using a random grid in WBS(2), are there any benefits in doing so? In my view, the lack of exact reproducibility can also be seen as an advantage. If the speed of execution is not an issue (e.g., for shorter datasets), the user can take advantage of the randomness of WBS(2) and execute the WBS(2).SDLL procedure multiple times and consider the empirical distribution of the detected change-points across the runs. The frequency (across the runs) with which a given change-point is being detected can then be seen as a measure of its significance/importance. Such considerations are not available with a fixed grid. This way of thinking is not dissimilar to the idea behind stability selection (Meinshausen and Bühlmann 2010).

*WBS2.SDLL in misspecified models.* Both Lund and Shi and Seo query the viability of WBS2.SDLL when applied to data that follow, or suggest, stochastic models other than the simple piecewise-constant signal plus noise. Lund and Shi ask if “more parsimonious” descriptions of teeth-like data (e.g., via a time series model with a seasonal component) may offer a superior alternative to the model returned by WBS2.SDLL, but I believe that this depends on the definition of parsimony used. The WBS2.SDLL routine as defined in the paper takes three parameters only: $\tilde{M}$, $\tilde{\zeta }_T$ and $\beta$, and from this point of view a model returned by WBS2.SDLL can hopefully be seen as parsimonious. One does not have to parameterise the signal returned by WBS2.SDLL by the locations of the detected change-points, which may indeed not be parsimonious if the number of change-points is large.

Seo applies WBS2.SDLL under model misspecification: to a random walk path and to a realisation of a (stationary) SETAR model, and obtains frequent change-points. My belief is that applying a reliable change-point detection procedure may be beneficial to the analyst even in some situations in which the model is obviously misspecified. An an example, consider the time series of daily COVID-19 associated deaths in the UK, starting from 6th March 2020 and last accessed on 6th August 2020. This is shown in the left plot of Fig. 1, together with the corresponding WBS2.SDLL fit. One fairly obvious observation is that quite possibly, WBS2.SDLL is not appropriate as a modelling device for this dataset because it over-reacts to the visually apparent seasonality in the data, from time 30 or 35 onwards. However, there seem to be some benefits of applying WBS2.SDLL even in this misspecified context. To give two examples of possible benefits of the WBS2.SDLL fit, we firstly observe that it suggests that the pattern of daily deaths was increasing up to time 30 or 35, which may be an interesting observation from the data-analytic point of view. A visual inspection of the raw data does not necessarily suggests this. Secondly, we would argue that the WBS2.SDLL fit brings out quite cleanly one of the seasonal patterns in the latter part of the data, which again is less obvious from the visual inspection of the raw counts.

**Fig. 1:**
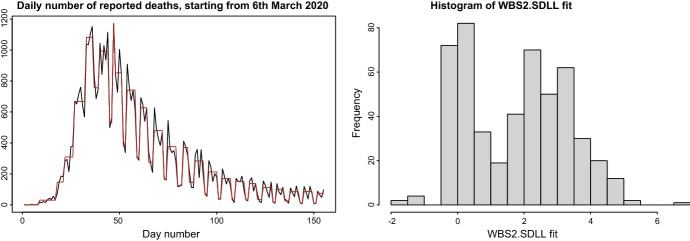
Left: daily number of COVID-19-associated deaths in the UK, starting from 6th March 2020 (black), together with the corresponding WBS2.SDLL fit (brown). Right: histogram of the values of the WBS2.SDLL fit to a sample path from Seo’s SETAR model

We also revisit the SETAR example from Seo’s contribution. Given the data (only), it may not be obvious to the analyst that the SETAR model is the most appropriate one, and a WBS2.SDLL fit may play a useful role in arriving at the right model. The right plot of Fig. 1 shows the histogram of the values of the WBS2.SDLL fit (over time $t = 1, \ldots , 500$) to a sample path simulated from Seo’s SETAR model. The clear bimodality in the empirical distribution of the WBS2.SDLL values may in fact be helpful in suggesting the SETAR model to the analyst, as it (correctly) indicates that the data-generating process may oscillate between two signal values. We note that a similar bimodality is completely visually absent from the raw data values themselves, which shows a good “signal extraction” power of WBS2.SDLL.

*WBS2 vs Seeded BS.* I view Seeded BS as a very interesting development, although I cannot help thinking of it as a rather close neighbour of WBS. In particular, I would be surprised if WBS2 on a deterministic grid ended up being much different in practical terms from Kovács et al.’s ASeedBS, especially if both methods used all available intervals when operating on short current segments of the data. Furthermore, just like Seeded BS, WBS2 can also be combined with the NOT solution path: in this construction, the CUSUMs targeted at each stage of the procedure will not be the largest available ones, but those that live on the shortest possible data intervals but are large enough to exceed a prescribed threshold.

*WBS2 vs MOSUM.* I appreciate Cho and Kirch’s comparison of the WBS2 and MOSUM approaches. One possible issue with MOSUM as defined in their contribution is that there may not be a single parameter value for which it works well across all scenarios. MOSUM with $\alpha = 0.1$ may work well for no or infrequent change-point scenarios, while $\alpha =0.9$ may work well for frequent change-point ones. By contrast, SDLL is specifically constructed to work well in both infrequent and frequent change-point settings without manual fine-tuning. It would of course be interesting to see to what extent it is possible to select regularity parameters such as the $\alpha$ in the MOSUM procedure automatically from the data.

*WBS2.SDLL for heteroskedastic errors/robust WBS.SDLL.* Banerjee asks whether it would be possible to develop a version of WBS2 applicable to heteroskedastic errors. We have coded up, and made available on https://github.com/pfryz/wild-binary-segmentation-2.0/blob/master/WBS2_SDLL_robust.R, a version of WBS2.SDLL that targets change-points in the piecewise-constant median of the data. The model is now

$$\begin{aligned} X_t = f_t + \varepsilon _t,\quad t = 1, \ldots , T, \end{aligned}$$

where $\varepsilon _t$ has median zero and is serially independent, but needs no moment conditions and can be arbitrarily heterogeneous. The key difference compared with the non-robust WBS2.SDLL is that on any data interval $(X_s, \ldots , X_e)$, we work with the CUSUM of $(\text {sign}\{X_s - \text {median}(X_{s:e})\}, \ldots , \text {sign}\{X_e - \text {median}(X_{s:e})\})$, rather than with the CUSUM of $(X_s, \ldots , X_e)$ itself.

*WBS2.SDLL in correlated error settings.* Lund and Shi ask whether WBS2.SDLL can be modified to work in autocorrelated error settings. One obvious idea might be to replace the threshold values as defined in the paper by ones based on the estimated long-run standard deviation of the innovation process, e.g., as recently done in the context of a different method in Dette et al. (2018). However, this would not resolve the issue in the case in which the autocorrelation structure itself might change, a setting we plan to consider in our future work on the topic.

*Performance of WBS2.SDLL for no-change-point scenarios.* Lund and Shi point out that the percentage of times that WBS2.SDLL correctly detects no change-points in constant signal settings seems to be lower than the advertised 90 or 95%. This agrees with our observation, and is something we did not realise while working on the original paper. To remedy this, we suggest using the thresholds developed by Fang et al. (2020), which asymptotically guarantee the exact probabilities. Alternatively, we refer the reader to a simpler result formulated in Anastasiou and Fryzlewicz (2018), which states that in the Gaussian constant signal case, with probability converging to one, absolute CUSUMs of all sub-intervals of the data are bounded by $\sigma (1 + \delta ) \sqrt{3 \log \, T}$ for any $\delta > 0$.

*Consistency proof for WBS2.SDLL.* Lund and Shi mention that the consistency proof is not easy to read. Under the present space constraints, it would be impossible for us to provide a more complete proof, but we would like to point out that the proof techniques are pretty standard and very close to those in Fryzlewicz (2014) and Baranowski et al. (2019), where some of the arguments used are elaborated in more detail. If the distances between change-points are of order $\asymp T$, then their number cannot increase with *T*, but their locations do not have to be fixed in rescaled time. We use in-fill asymptotics, as do many other related works. The jump sizes are permitted to tend to zero at the request of a referee.

*WBS2.SDLL vs PELT and other dynamic programming techniques.* Lund and Shi ask: “if the variance parameter $\sigma ^2$ were known, what would stop us from using PELT, FPOP, or another related dynamic programming technique with a penalized likelihood for rapid computation?” I am not aware of a penalised likelihood approach with a fixed penalty that works well in both frequent and infrequent change-point settings. In particular, my experience with the BIC penalty is that it tends to work well for infrequent change-points, but not for frequent ones, as reported in the original paper. It is possible that less aggressive penalties, such as AIC, might work well in frequent change-point models, but AIC is known to be theoretically inconsistent. Adaptive penalty approaches, as reviewed in the paper, may help here but they are not necessary computationally efficient, and some suffer from other issues: see the original paper for details.

*WBS2.SDLL in models other than piecewise constant.* Banerjee asks whether WBS2 can be useful in models other than piecewise constant, such as piecewise linear. It is perfectly possible to equip NOT (Baranowski et al. 2019) with a WBS2-like mechanism for drawing intervals, which would result in a more through exploration of the space of intervals in comparison with the older WBS-like mechanism used in Baranowski et al. (2019). Our guess is that SDLL could still be used for model selection in settings such as piecewise linear (based on the largest contrasts suitable for the piecewise linear model). However, as explained in Baranowski et al. (2019), such largest non-CUSUM contrasts would not be able to estimate the change-points locations. Therefore, we believe that it would be a possibility to estimate the number of change-points in the piecewise-linear model via WBS2.SDLL, and then their locations via NOT. Such a hybrid procedure may well lead to improved model selection in the piecewise linear and other piecewise polynomial models.

*Other points, thanks and conclusion.* I would like to conclude by thanking all contributors for their warm and encouraging words about the new proposal. It was very interesting for me to see that SDLL may be applicable more widely: it appeared to be working well also in the Seeded BS and MOSUM contexts. Finally, I found the JFNL estimator of $\sigma$, proposed in Kovács et al. very appealing, and I am keen to try it out in my future work.

## References

[CR1] Anastasiou, A., Fryzlewicz, P. (2018). Detecting multiple generalized change-points by isolating single ones. Preprint 10.1007/s00184-021-00821-6 PMC8142888 34054146

[CR2] Baranowski R, Chen Y, Fryzlewicz P (2019). Narrowest-over-threshold detection of multiple change-points and change-point-like features. Journal of the Royal Statistical Society Series B.

[CR3] Cho H, Fryzlewicz P (2012). Multiscale and multilevel technique for consistent segmentation of nonstationary time series. Statistica Sinica.

[CR4] Dette, H., Schüler, T., Vetter, M. (2018). Multiscale change point detection for dependent data. Preprint.

[CR5] Fang X, Li J, Siegmund J (2020). Segmentation and estimation of change-point models: false positive control and confidence regions. Annals of Statistics.

[CR6] Fryzlewicz P (2014). Wild Binary Segmentation for multiple change-point detection. Annals of Statistics.

[CR7] Kovács, S., Li, H., Bühlmann, P. (2020) Seeded Binary Segmentation: A general methodology for fast and optimal change point detection. Preprint.

[CR8] Meinshausen N, Bühlmann P (2010). Stability selection. Journal of the Royal Statistical Society.

